# Relationship of PD-1 (PDCD1) and PD-L1 (CD274) Single Nucleotide Polymorphisms with Polycystic Ovary Syndrome

**DOI:** 10.1155/2021/9596358

**Published:** 2021-01-04

**Authors:** Rui Han, Xiaoyun Gong, Yuejie Zhu, Xiaoran Liu, Yan Xia, Yuhong Huang, Meng Zhang, Yunian Zhang, Xiaolin La, Jianbing Ding

**Affiliations:** ^1^Department of Prenatal Diagnosis, Reproductive Medicine Center, The First Affiliated Hospital of Xinjiang Medical University, Urumqi, 830011 Xinjiang, China; ^2^Department of Immunology, College of Basic Medicine, Xinjiang Medical University, Urumqi, 830011 Xinjiang, China; ^3^State Key Laboratory of Pathogenesis, Prevention, Treatment of Central Asian High incidence Diseases, The First Affiliated Hospital of Xinjiang Medical University, Urumqi, 830011 Xinjiang, China; ^4^Department of Reproductive Assistance, Center for Reproductive Medicine, The First Affiliated Hospital of Xinjiang Medical University, Urumqi, 830011 Xinjiang, China

## Abstract

This study is to investigate the relationship of programmed cell death 1 (PD-1; also known as PDCD1) and programmed death-1-ligand 1 (PD-L1; also known as CD274) single nucleotide polymorphisms (SNPs) with polycystic ovary syndrome (PCOS). This study enrolled 330 PCOS patients and 350 matched controls. ELISA was used to detect the PD-1 and PD-L1 levels in serum. SnaPshot genotyping was performed to analyze the PD-1 and PD-L1 genotyping. Linkage disequilibrium and haplotype of TagSNP loci of PD-1 and PD-L1 genes were also detected. The relationship of genotypes and alleles with PCOS was analyzed. The levels of PD-1 and PD-L1 in the serum of PCOS patients were significantly lower than those in the control group (*P* < 0.01). The haplotype TT of PD-1 gene at rs10204525 and rs7421861 loci was significantly lower in the PCOS group than in the control group (*P* < 0.001, OR = 0.67, and 95%CI = 0.54‐0.84). PD-L1 gene SNP loci rs2282055, rs2890658, rs10125854, and rs702275 had linkage disequilibrium. The haplotypes TAAA, GAAC, GAGC, GCAA, and TCGA of PD-L1 gene SNP loci were significantly higher in PCOS patients than in the control group, whereas haplotypes GAAA, TAAC, TCAA, GCGA, GCAC, and TCGC of PD-L1 gene SNP loci were significantly lower in PCOS patients than in the control group. PD-1 and PD-L1 SNPs may be related to the pathogenesis of PCOS. PD-1 gene SNP loci rs10204525 and rs7421861 and PD-L1 gene SNP loci rs2282055, rs2890658, rs10125854, and rs702275 may be new candidate polymorphic loci for PCOS.

## 1. Introduction

Polycystic ovary syndrome (PCOS) is a metabolic disease of endocrine disorders in women. Its incidence in women of childbearing age is 5%-10% [1–3]. PCOS is mainly manifested as abnormal menstruation, infertility, obesity, hairy, polycystic ovarian changes, hyperandrogenemia, insulin resistance, and induced hyperinsulinemia [4]. With the changes in people's lifestyle and environment, the incidence of PCOS has been increasing year by year [5]. At present, the pathogenesis of PCOS is still unclear. Epidemiological survey reveals obvious family clustering characteristics of PCOS [6]. Chen et al. have reported 11 susceptible genes related to PCOS disease and extensively tested them in European and American populations [7]. Therefore, genetic factors play a very important role in the etiology of PCOS.

Programmed cell death 1 (PD-1; also known as PDCD1) and programmed death-1-ligand 1 (PD-L1; also known as CD274) play important negative regulatory roles in inflammation, autoimmune diseases, and tumors. They are widely involved in suppressing inflammatory response and tumor immune evasion [8, 9]. Genetic mutations in PD-1 and PD-L1 not only increase the risk of kidney cancer [10] and thyroid cancer [11], but also are closely related to the course, clinical stage, and metastasis of the tumor and even affect the function of kidney transplantation [12]. In a multicenter meta-analysis by Mohammad et al. [13], it is found that PD-1 and PD-L1 gene polymorphisms were related to susceptibility and progression of all cancers. It is reported that PD-1 and PD-L1 gene polymorphisms can affect the susceptibility to ankylosing spondylitis [14, 15]. In addition, PD-L1 gene polymorphisms are reported to be associated with lung cancer susceptibility [16, 17]. Therefore, PD-1 and PD-L1 gene polymorphisms are closely related to the occurrence of diseases such as cancer. However, whether the PD-1 and PD-L1 gene polymorphisms are related to the occurrence and development of PCOS has not been reported.

In this study, we explored the correlation between PD-1 and PD-L1 gene polymorphisms and the pathogenesis of PCOS.

## 2. Materials and Methods

### 2.1. Patients

This study included 330 patients diagnosed with PCOS at the Reproductive Fertility Center of the First Affiliated Hospital of Xinjiang Medical University. Meanwhile, 350 non-PCOS women of childbearing age who matched the clinical indicators of PCOS patients were recruited as control group. The inclusion criteria for the PCOS patient group included the following: (1) the diagnosis of PCOS met the diagnostic criteria established by the Rotterdam International Conference [18]. Patients with any two items of the following three items can be diagnosed: little or no ovulation, hyperandrogenemia, ovarian volume > 10 mL on ultrasound imaging, and ≥12 follicles with 2-9 mm in diameter. (2) The patients did not take drugs that may affect hormones, blood glucose, or insulin at 3 months before the blood collection. (3) The patients did not implement planned weight loss. Exclusion criteria for patients with PCOS included the following: (1) patients with congenital adrenal hyperplasia, Cushing syndrome, and ovarian or adrenal tumors; (2) patients with hyperprolactinemia, thyroid disease, or pituitary tumor; (3) patients with severe primary diseases such as cardiovascular disease, liver disease and kidney disease, or mental illness; (4) patients with pregnancy or suspicious pregnancy, lactation, suffering from other endocrine diseases, with long-term oral contraceptives, or subcutaneous implanted contraception. The inclusion criteria of the control group included the following: (1) patients with infertility due to tubal obstruction or male reasons. (2) Their age, body mass index, and other clinical indicators were matched with those in the PCOS group. (3) They had regular menstruation and normal serum androgens. (4) They had no clinical hyperinsulinemia, no symptoms of hyperandrogenemia, and no clinical manifestations of PCOS such as hairy, acne, and skin seborrhea. Written informed consent was obtained from every patient, and the study was approved by the Ethics Committee of the First Affiliated Hospital of Xinjiang Medical University.

### 2.2. Detection of Clinical Indicators

We recorded the clinical information of all patients and normal controls, including age, weight, height, waist circumference, and hip circumference. Body mass index was calculated as BMI = weight (kg)/height^2^ (m^2^). Waist-to-hip ratio (WHR) was calculated as waist circumference/hip circumference. Peripheral blood (3-5 mL) was collected from patients and controls. The serum levels of follicle-stimulating hormone (FSH), luteinizing hormone (LH), and testosterone (T) were measured using a chemiluminescence immunoassay analyzer (i2000SR, Abbott). Fasting blood glucose (FBG) was measured using hexokinase method (AU5821, Beckman, Germany). Fasting insulin (FNS) was detected by chemiluminescence method (i2000SR, Abbott). The formula for calculating the insulin resistance index (HOMA-IR) is as follows: [HOMA − IR = fasting insulin (mIU/L) × fasting blood glucose (mmol/L)/22.5].

### 2.3. ELISA

The serum levels of PD-1 and PD-L1 were detected with corresponding ELISA kits (E-EL-H1534c/E-EL-H1547c Elabscience Biotechnology Co. Ltd, Wuhan, China) according to the kit instructions. OD450 was measured, and the concentrations of PD-1 and PD-L1 in the specimens were calculated by the standard curve.

### 2.4. DNA Extraction and Genotyping

**Figure 1:**
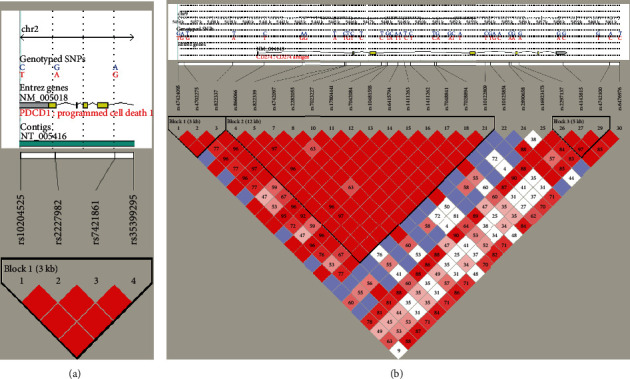
Structural map of linkage disequilibrium of SNPs in HapMap. (a) HapMap shows the linkage disequilibrium structure of PD-1 gene SNPs. (b) HapMap shows the linkage disequilibrium structure of PD-L1 gene SNPs.

DNA was extracted from peripheral blood using TIANamp Blood DNA Kit (TIANGEN, Beijing, China). Data on the linkage disequilibrium between PD-1 and PD-L1 gene single nucleotide polymorphisms (SNPs) in the Chinese population were obtained by querying the NCBI database. By setting the linkage disequilibrium coefficient *r*^2^ of SNP loci ≥ 0.8 and the minimum allele frequency > 5% as the standard, we used the Haploview software to select three TagSNP loci representing the four SNP loci of the entire PD-1 gene and 12 TagSNP loci representing 24 SNP loci for the entire PD-L1 gene. Finally, the HapMap map was generated (Figure 1).

### 2.5. SnaPshot Typing Test

By using the SnaPshot typing technique [19], we detected a total of 15 SNP loci in PD-1 and PD-L1 genes. The sequences of PD-1 and PD-L1 were retrieved through Gene Bank. The primers were designed using the Primer 5.0 software and synthesized by Shanghai Sangon Biotech (China). The primer sequences are shown in Table 1. Briefly, after PCR amplification of the genomic DNA sample, the amplified PCR product (2 *μ*L) was purified and mixed with 1 *μ*L SnaPshot Mix and 0.2 *μ*L primers, and then, the reaction system was supplemented with ddH_2_O to 6.0 *μ*L to perform PCR reaction. Then, 1 *μ*L of the product was taken, and 9 *μ*L of the loading Hi-Di was added. The samples were denatured at 95°C for 3 min, and an ice-water bath was performed immediately. After that, the sample was added with Hi-Di Formamide 9 *μ*L, denatured at 95°C for 5 min, and then sequenced with an ABI3730XL sequencer. GeneMapper4.1 (AppliedBiosystems Co. Ltd, USA) was used to analyze the data.

### 2.6. Statistical Analysis

SPSS17.0 was used to analyze the data. The data was expressed as mean ± SD. The Hardy-Weinberg (H-WE) equilibrium was used to detect the genotype frequency distribution of TagSNP loci of the PD-1 gene and PD-L1 gene in the PCOS group and the control group, respectively. SHEsis (http://analysis.bio-x.cn/myAnalysis.php) was used to analyze the linkage disequilibrium and haplotype of the PD-1 gene SNP loci rs7421861 and rs10204525 and PD-L1 gene SNP loci rs2282055, rs2890658, rs702275, and rs10125854. The independent t test was used to compare the data between the two groups. Chi-square test was used to compare the genotype and allele frequency, and the correlation was analyzed by logistic regression [20]. The OR value and 95% confidence interval were calculated. *P* < 0.05 was considered statistically significant.

## 3. Results

### 3.1. Clinical Information of PCOS Group and Control Group

A total of 330 patients with PCOS and 350 controls were included in this study. They were all from Xinjiang Uygur Autonomous Region, China. They were aged 20-39 years. The clinical data of included subjects are shown in Table 2. There was no statistically significant difference in age, BMI, WHR, and FSH between PCOS group and control group (*P* > 0.05). Statistical results showed that the levels of T, LH, LH/FSH, FBG, FNS, and HOMA-IR in the PCOS group were significantly higher than those in the control group (Table 2, *P* < 0.05). These results indicate that the PCOS group has significant clinical differences compared with the normal control group.

### 3.2. Serum PD-1 and PD-L1 Level in Patients

**Figure 2:**
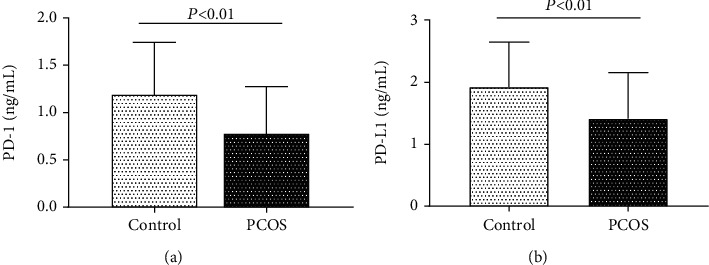
Serum levels of PD-1 and PD-L1 in PCOS and control groups. (a) PD-1 levels in the serum of PCOS group and control group. (b) PD-L1 levels in the serum PCOS and control groups.

ELISA was conducted to measure the serum levels of PD-1 and PD-L1. The results showed that the serum levels of PD-1 (Figure 2(a)) and PD-L1 (Figure 2(b)) in patients with PCOS were 0.76 ± 0.03 ng/mL and 1.41 ± 0.04 ng/mL, which were significantly lower than those in the control group (1.19 ± 0.03 ng/mL and 1.93 ± 0.04 ng/mL, respectively) (*P* < 0.01), suggesting that abnormalities of PD-1 and PD-L1 in the serum of PCOS patients may be related to the onset of PCOS.

### 3.3. PD-1 and PD-L1 Gene SNPs' H-WE Test

The SnaPshot typing results were shown in supplementary file 1. H-WE test was used to detect the gene frequency distribution of PD-1 gene and PD-L1 gene TagSNP loci in PCOS group and control group, respectively. The results showed that the genotype distribution of the TagSNP loci of the PD-1 gene and PD-L1 gene in the PCOS group and the control group was consistent with the H-WE with good agreement (Table 3, *P* > 0.05). Thus, these genotypes can be used for subsequent haplotype analysis.

### 3.4. SNPs of PD-1 and PD-L1 in Patients

Through HapMap analysis, we obtained 15 TagSNP loci of the PD-1 gene and PD-L1 gene. Among them, the allele frequency of the rs7421861 and rs10204525 in the PD-1 gene SNP loci and the rs2282055, rs2890658, rs702275, and rs10125854 in the PD-L1 SNP loci gene had statistically significant differences between the PCOS group and the control group (Table 4, *P* < 0.05). These results indicate that the SNPs of PD-1 gene and PD-L1 gene might be related to the pathogenesis of PCOS disease.

In addition, we found that the 15 TagSNP loci of PD-1 gene and PD-L1 gene in the PCOS group and the control group all conformed to H-WE. At the same time, by using the SHEsis software, we performed linkage disequilibrium and haplotype analyses for the six SNP sites with statistical differences in Table 4, including rs7421861 and rs10204525 of the PD-1 gene SNP loci and the rs2282055, rs2890658, rs702275, and, rs10125854 of the PD-L1 gene SNP loci. The results showed that the haplotype TT of rs7421861 and rs10204525 of the PD-1 SNP loci had a haplotype frequency of 0.45 in the control group and of 0.36 in the PCOS group. The TT haplotype distribution frequency in the PCOS group was significantly lower than that in the control group (Table 5; OR = 0.67, 95%CI = 0.54‐0.84, and *P* < 0.05). Moreover, in the PCOS group and the control group, there were significant differences in the haplotypes GAAA, TAAA, GAAC, TAAC, GAGC, GCAA, TCAA, GCGA, TCGA, GCAC, and TCGC of the rs2282055, rs2890658, rs702275, and rs10125854 SNP loci of the PD-L1 gene (Table 6, *P* < 0.05). The above results suggest that the haplotypes of the SNP loci of PD-1 and PD-L1 genes are related to the occurrence and development of PCOS disease.

## 4. Discussion

Chronic inflammation has been a uniform clinical feature in patients with PCOS. The PD-1, a member of the B7/CD28 costimulatory molecule family, is a cosuppression signal molecule that inhibits T cell activation [21]. PD-L1, PD-1 ligand, can induce T cell apoptosis and stimulate cells to secrete IL-10, thereby mediating immunosuppressive effects [22]. The physiological function of PD-L1 is to regulate the inflammatory response and limit tissue damage. Thus, the interaction between PD-1 and PD-L1 can prevent excessive inflammation and suppress the immune response.

In this study, we tested the levels of PD-1 and PD-L1 in serum. The results showed that compared with the control group, the levels of PD-1 and PD-L1 were lower in the serum of PCOS patients, which is consistent with the study of Ni et al. [23]. The low levels of PD-1 and PD-L1 in the serum of PCOS patients may be related to their inability to effectively inhibit T and B cell function and proliferation, thus leading to the pathogenesis of PCOS. However, whether the decrease in PD-1 and PD-L1 levels can be used as an immunological diagnosis and treatment index for PCOS disease requires further research.

Mounting evidence suggests that PCOS might be a complex multigenic disorder strongly influenced by epigenetic and environmental factors, including diet and lifestyle factors [24]. Pedigree studies have shown that PCOS has significant family clustering characteristics [25, 26]. At present, the largest twin studies in PCOS have found that the incidence of PCOS was twice as high in monozygotic twins as in dizygotic twins, indicating that genetic factors play a very important role in PCOS pathogenesis [27]. In this study, we used SnaPshot typing to detect PD-1 and PD-L1 gene polymorphisms in PCOS and analyzed the correlation between PD-1 and PD-L1 gene polymorphisms and PCOS. The results showed that the allele frequencies of the PD-1 gene SNP loci (rs7421861 and rs10204525) and PD-L1 gene SNP loci (rs2282055, rs2890658, rs702275, and rs10125854) were significantly different between the two groups, indicating that these SNP loci may be related to the development of PCOS.

In addition, in order to investigate whether the frequency distribution of genes and genotypes of PCOS patients and control groups has reached genetic balance and to understand whether they are representative of the population, we use the H-WE to detect the genotype frequency distribution of PD-1 gene SNP loci rs7421861 and rs10204525 and PD-L1 gene SNP loci rs2282055, rs2890658, rs702275, and rs10125854. H-WE test can reflect the representative of the population [28, 29]. The results confirmed that these SNP loci conformed to H-WE. These results showed that these SNP loci were not only significantly different between the PCOS group and the control group, but also were representative of the population.

In this study, we analyzed the linkage disequilibrium and haplotype between SNP loci of the PD-1 gene. The results showed that there was a linkage disequilibrium between rs10204525 and rs7421861 of the PD-1 gene SNP loci. At the same time, the haplotype TT of PD-1 gene at rs10204525 and rs7421861 loci was less frequently distributed in the PCOS group than in the control group. It is reported that PD-1 gene polymorphism is related to some inflammatory diseases and autoimmune diseases, such as allergic asthma [30]. The PD-1 gene polymorphism rs2227981 is associated with tumor occurrence, including digestive system tumors and female-specific tumors [31]. PD-1 polymorphism is associated with susceptibility to diseases such as gastric cancer, clear cell renal cancer, HBV, and HCV [32–34]. After the PD-1 gene is knocked out, its original negative regulatory effect would be lost, resulting in increased levels of inflammatory factors, which promotes the imbalance of proinflammatory and anti-inflammatory cytokines and thereby promotes the occurrence of related diseases [35]. PD-1 is also related to immune dysfunction [36]. Moreover, inflammation is one of the clinical features of PCOS women [37]. Thus, we believe that the immune response involving PD-1 gene polymorphism is related to the pathogenicity of PCOS. When the T allele and T allele of PD-1 gene are mutated at the same time, haplotype TT is a protective factor for the occurrence of PCOS disease. The haplotype of PD-1 gene is related to the occurrence and development of PCOS disease, which indicates that the PD-1 gene polymorphism is related to the pathogenesis of PCOS.

In the linkage disequilibrium and haplotype analysis of the PD-L1 gene SNP loci rs2282055, rs2890658, rs10125854, and rs702275, the results showed that there was a linkage disequilibrium between the PD-L1 gene SNP loci rs2282055, rs2890658, rs10125854, and rs702275. And the haplotype TAAA, GAAC, GAGC, GCAA, and TCGA frequencies in the PCOS group were significantly higher than the control group. However, the haplotype GAAA, TAAC, TCAA, GCGA, GCAC, and TCGC frequencies were significantly lower in the PCOS group than in the control group. Studies have reported that the PD-L1 gene rs4143815 may be associated with an increased risk of gastric cancer [38, 39] and non-small-cell lung cancer [40, 41], which may be caused by increased PD-L1 expression and suppression of immune tumor surveillance [42]. Women with PCOS have an increased risk of endometrial cancer, ovarian cancer, endocrine adenocarcinoma, pancreatic cancer, and other cancers [43]. We believe that the polymorphism of the PD-L1 gene affects the expression of PD-L1, which in turn promotes the onset of PCOS. These results suggest that haplotypes TAAA, GAAC, GAGC, GCAA, and TCGA might be susceptibility factors for the occurrence of PCOS, while haplotypes GAAA, TAAC, TCAA, GCGA, GCAC, and TCGC might be protective factors for the occurrence of PCOS. The haplotype of PD-L1 gene SNP loci is related to the occurrence and development of PCOS which indicates that the polymorphism of PD-L1 gene is related to the pathogenesis of PCOS.

However, there are still some limitations in the study. First, the number of cases in this study is limited. Second, the specific pathways and mechanisms of PD-1 gene and PD-L1 gene in the pathogenesis of PCOS were not investigated. Further studies with large sample size are still needed to reveal the underlying mechanisms.

## 5. Conclusions

In summary, in this study, we reported for the first time that PD-1 gene and PD-L1 gene may be involved in the pathogenesis of PCOS. Our results showed that the genotype distribution, allele frequency, and haplotype of the PD-1 gene SNP loci rs10204525 and rs7421861 and PD-L1 gene rs2282055, rs2890658, rs10125854, and rs702275 were all related to the pathogenesis of PCOS. In the follow-up studies, we will further explore the correlation of PD-1 and PD-L1 gene polymorphisms with the clinical characteristics of PCOS. These results will help screen and protect susceptible populations at the genetic level, will also provide theoretical basis for individualized diagnosis and treatment programs for PCOS patients, and will provide a basis for developing novel biological markers for early screening and diagnosis of PCOS.

## Figures and Tables

**Table 1:** SNP sites and primer sequences of PD-1 gene and PD-L1 gene.

SNP sites	Polymorphism	Primers	Product length	Extension	Products	Extension primer
rs10204525	(A/G)	F: CAGGGAAGCTGAGGCAGTAA R: TTTCAGGAATGGGTTCCAAG	272 bp	Forward	(A/G)	TTTTTTACCTAGGGCCCCCCAT
rs4742095	(G/T)	F: CCCACAGCCCTTCTTGTGAA R: TCCAGAGTAGGATACTATAGCCAGT	277 bp	Reverse	(A/C)	GGCAGTATGTTGACAGTATTTCAAAG
rs702275	(G/T)	F: TCTTCCTTGATTTCCTTTTATCCA R: GCAATGAAAAGCCACCAGAT	236 bp	Reverse	(A/C)	TTTTTTGAAAATCTTTAGGAGCTGTTTCTGTAG
rs822339	(C/T)	F: TAACTCTGGCCCAAGGAAAA R: TTTTGGTCTGTTTATGTCACTGG	250 bp	Reverse	(A/G)	TTTTTTTTTTTTTTTCATCTACAGGATAGACGGAAAGGA
rs2282055	(A/C)	F: TGTAGGGGGAAAAAGCATTG R: GCCCACAGCCACATAAACTT	258 bp	Reverse	(G/T)	TTTTTTTTTTTTTTTTTTTTTTGTCAGATTCTCCTTGCTCTGAAAT
rs6415794	(A/T)	F: CCATCAATCTGAGGGCTAACA R: GATGTCATGAATGGAGAAGCA	264 bp	Forward	(A/T)	TTATGTTGTATTTCTGGTCCCTGAT
rs2890658	(A/C)	F: CCTGGGCAACAGAGAAAGAC R: GGAATGGCGAGATACCTGTG	306 bp	Forward	(A/C)	TTTTTTAAGAGGAAGTGAAATAATCAAGGC

**Table 2:** Clinical characteristics of PCOS patients and control groups.

Items	PCOS (*n* = 330)	Control (*n* = 350)	*t*	*P*
Age	25.82 ± 3.56	26.23 ± 3.41	-1.42	0.08
BMI	23.90 ± 2.95	23.50 ± 2.17	1.75	0.09
WHR	0.77 ± 0.16	0.75 ± 0.16	1.61	0.11
FSH (IU/L)	6.80 ± 2.00	6.58 ± 1.81	1.53	0.45
T (nmol/L)	1.23 ± 0.70	0.72 ± 0.46	11.49	0.01^∗^
LH (IU/L)	11.95 ± 4.96	9.12 ± 2.90	9.10	0.02^∗^
LH/FSH	1.75 ± 0.83	1.35 ± 0.50	7.59	0.03^∗^
FBG (mmol/L)	5.40 ± 0.67	5.01 ± 0.46	9.03	0.03^∗^
FNS (mIU/L)	13.58 ± 5.82	8.67 ± 4.31	12.48	<0.01^∗^
HOMA-IR	3.27 ± 1.49	1.93 ± 1.00	13.73	0.01^∗^

Note: PCOS: polycystic ovary syndrome; BMI: body mass index; WHR: waist hip ratio; FSH: follicle-stimulating hormone; LH: luteinizing hormone; T: testosterone; FBG: fasting blood glucose; FNS: fasting insulin; HOMA-IR: insulin resistance index. Data were shown as mean ± SD; ^∗^*P* < 0.05.

**Table 3:** H-WE equilibrium test of genotype distribution of SNP loci in PD-1 and PD-L1 genes.

Gene	TagSNP	PCOS		Control	
		*χ* ^2^	*P*	*χ* ^2^	*P*
PD-L1 gene	rs6415794	0.95	0.33	3.18	0.07
	rs822339	1.50	0.22	1.36	0.24
	rs17804441	1.53	0.22	0.19	0.66
	rs2282055	1.59	0.21	0.18	0.67
	rs4143815	1.30	0.26	1.41	0.24
	rs2890658	3.54	0.06	1.69	0.20
	rs2297137	0.15	0.70	2.44	0.12
	rs10125854	0.00	0.98	0.95	0.33
	rs6476976	1.53	0.22	3.20	0.07
	rs702275	2.57	0.11	1.41	0.24
	rs16923173	2.93	0.09	3.33	0.07
	rs4742095	0.61	0.43	2.97	0.09
PD-1 gene	rs7421861	0.52	0.48	2.97	0.09
	rs2227982	0.14	0.71	1.28	0.26
	rs10204525	0.25	0.62	1.80	0.18

Note: PCOS: polycystic ovary syndrome; SNP: single nucleotide polymorphism. *P* > 0.05 is considered as good agreement with H-WE equilibrium test.

**Table 4:** Allele frequency distributions of PD-L1 and PD-1 gene SNP loci in the PCOS and control groups.

Gene	SNP	Allele	PCOS (*n* = 330)	Control (*n* = 350)	*χ* ^2^	*P*
PD-L1 gene	rs6415794	T A	456 (69.05) 204 (30.95)	469 (66.96) 231 (33.04)	0.68	0.41
	rs822339	C T	424 (64.29) 236 (35.71)	420 (60.00) 280 (40.00)	2.65	0.10
	rs17804441	A G	447(67.71) 213 (32.29)	486 (69.42) 214 (30.58)	0.46	0.49
	rs2282055	C A	348 (52.67) 312 (47.33)	433 (61.88) 267 (38.12)	11.77	<0.01^∗^
	rs4143815	C G	468 (70.98) 192 (29.02)	489 (69.86) 211 (30.14)	0.20	0.65
	rs2890658	A C	440 (66.67) 220 (33.33)	430 (61.45) 270 (38.55)	4.01	0.04^∗^
	rs2297137	G A	474 (71.88) 186 (28.12)	473 (67.54) 227 (32.46)	3.02	0.08
	rs10125854	A G	406 (61.46) 254 (38.54)	47 1(67.25) 229 (32.75)	4.97	0.03^∗^
	rs6476976	C T	447 (67.71) 213 (32.29)	487 (69.57) 213(30.43)	0.55	0.46
	rs702275	T G	315 (47.77) 345 (52.23)	383(54.78) 317 (45.22)	6.68	0.01^∗^
	rs16923173	G A	438 (66.37) 222 (33.63)	449 (64.21) 251 (35.79)	0.70	0.40
	rs4742095	G T	583 (88.39) 77 (11.61)	606 (86.52) 94 (13.48)	1.08	0.30
PD-1 gene	rs7421861	T C	396 (59.97) 264 (40.03)	457 (65.22) 243 (34.78)	4.00	0.04^∗^
	rs2227982	T C	371 (56.25) 289 (43.75)	387 (55.22) 313 (44.78)	0.15	0.70
	rs10204525	T C	370 (56.10) 290 (43.90)	431 (61.59) 269 (38.41)	4.23	0.04^∗^

Note: PCOS: polycystic ovary syndrome; SNP: single nucleotide polymorphism. ^∗^*P* < 0.05.

**Table 5:** Haplotype frequency distribution of rs7421861 and rs10204525 of PD-1 gene.

Haplotype	Control (frequency)	PCOS (frequency)	*χ* ^2^	*P*	OR (95% CI)
CC	129.99 (0.19)	130.97 (0.20)	0.36	0.55	
TC	113.49 (0.16)	133.23 (0.20)	3.61	0.06	
CT	138.85 (0.20)	158.76 (0.24)	3.54	0.06	
TT	317.67 (0.45)	237.04 (0.36)	12.64	<0.01^∗^	0.67 (0.54-0.84)

Note: PCOS: polycystic ovary syndrome. ^∗^*P* < 0.05.

**Table 6:** Haplotype frequency distribution of rs2282055, rs2890658, rs702275, and rs10125854 loci of the PD-L1 gene.

Haplotype	Control (frequency)	PCOS (frequency)	*χ* ^2^	*P*	*OR* (95% CI)
GAAA	123.87 (0.18)	44.38 (0.07)	35.22	<0.01^∗^	0.35 (0.24~0.50)
TAAA	38.56 (0.06)	127.81 (0.19)	64.70	<0.01^∗^	4.35 (2.96~6.31)
GAAC	27.11 (0.04)	59.06 (0.09)	16.10	<0.01^∗^	2.54 (1.59~4.06)
TAAC	49.51 (0.07)	10.86 (0.02)	22.53	<0.01^∗^	0.23 (0.12~0.44)
GAGC	18.26 (0.03)	38.30 (0.06)	9.51	<0.01^∗^	2.39 (1.35~4.22)
GCAA	62.45 (0.09)	93.08 (0.14)	10.53	<0.01^∗^	1.75 (1.24~2.46)
TCAA	116.08 (0.17)	48.16 (0.07)	25.41	<0.01^∗^	0.41 (0.29~0.59)
GCGA	51.56 (0.07)	15.32 (0.02)	17.44	<0.01^∗^	0.31 (0.17~0.55)
TCGA	28.12 (0.04)	86.84 (0.13)	39.08	<0.01^∗^	3.78 (2.43~5.88)
GCAC	29.03 (0.04)	7.74 (0.01)	10.83	<0.01^∗^	0.28 (0.13~0.63)
TCAC	24.73 (0.03)	14.56 (0.02)	1.59	0.21	
TCGC	121.82 (0.17)	0.41 (0.00)	121.81	<0.01^∗^	0.00 (0.00~0.02)

Note: PCOS: polycystic ovary syndrome. ^∗^*P* < 0.05.

## Data Availability

The data that support the findings of this study are available on request from the corresponding author.

## Supplementary Materials

Supplementary Materials Supplementary file 1. The SnaPshot typing results.
